# High prevalence of HIV-1 transmitted drug resistance among therapy-naïve Burmese entering travelers at Dehong ports in Yunnan, China

**DOI:** 10.1186/s12879-018-3130-9

**Published:** 2018-05-08

**Authors:** Qicai Xuan, Shuwen Liang, Weihong Qin, Shuting Yang, A-mei Zhang, Ting Zhao, Hui Su, Zhiqing Xia, Binghui Wang, Xueshan Xia

**Affiliations:** 1 Faculty of Life Science and Technology, Kunming University of Science and Technology, Kunming, Yunnan China; 2 The First people’s hospital of Yunnan Province, Kunming, Yunnan China; 3 Care Center for International Travel Health in Yunnan, Kunming, Yunnan China; 4 Ruili Entry-Exit Inspection and Quarantine Authority, Ruili, Yunnan China; 5 Brighton College, 1 Eastern Road, Brighton, England UK

**Keywords:** Human immunodeficiency Virus-1, Transmitted drug resistance, Therapy-naïve

## Abstract

**Background:**

The overall success of Human immunodeficiency virus type 1 (HIV-1) antiretroviral therapy (ART) was heavily challenged upon the occurrence of drug resistance. Dehong Prefecture witnessed not only the first report of HIV-1 infection but also the experimental adoption of antiviral treatment in China. The transmission and epidemic of HIV-1 in Dehong is impacted by cross-border activities. The characteristics of HIV-1 drug resistance among therapy-naïve Burmese entering travelers in Yunnan and their speculated origin are still not clarified.

**Methods:**

Two hundred ninety-eight HIV-1 infected Burmese entering travelers at Dehong ports were recruited between 2003 and 2012. The partial HIV-1 *pol* gene fragments were amplified and sequenced for the analysis of drug-resistance mutations (DRMs). Phylogenetic analysis on *gag-pol* gene was conducted to elucidate phylogenetic and evolutionary characteristics of these drug resistant strains.

**Results:**

It was figured out that the occurrence ratio of HIV-1 drug resistance among HIV-1 infected entering travelers from Myanmar was up to 12.8%. The resistant mutations covered several types, including one type of PI mutations (L33F), six types of NRTI mutations and seven types of NNRTI. Close genetic relationship was observed in the phylogenetic analysis on *gag-pol* gene among the drug resistant strains respectively from Dehong, other Yunnan areas, neighboring provinces (Guangxi) and neighboring countries (Thailand and Myanmar).

**Conclusions:**

The findings in this study revealed that HIV drug resistant locus is spreading from the population who is receiving drug-resistance treatment to the new infectors, which indicates the urgency of surveillance work on drug resistance among the migrant population with high risks of HIV infection.

**Electronic supplementary material:**

The online version of this article (10.1186/s12879-018-3130-9) contains supplementary material, which is available to authorized users.

## Background

Antiretroviral therapy (ART) has greatly contributed to reducing HIV-1 viral load, prolonging patients’ lives, lowered the AIDS morbidity and mortality, and also prevented HIV-1 transmission [1–3]. However, HIV-1 drug resistance occured in a substantial proportion of treated patients and accumulated over time with the long-term use of ART [4]. Drug resistance has become the magnificent challenge for current antiretroviral therapy, especially in developing countries.

In the wake of implementation of ART, resistant strains carrying drug-resistant mutations have been increasingly prevalent [5]. The new infected individuals have greater chance to be infected directly by existing drug resistant strains instead of the natural generation of strains during the antiretroviral therapy procedure. The transmitted drug resistance (TDR), also known as acquired drug resistance, makes strains get more advantages in circuiting due to its insensitive response to various antiviral drugs. Hence, TDR has been a major difficulty and an intensive focus in AIDS treatment and HIV surveillance.

Yunnan province has been worst hit by HIV-1 in China [6]. The multi-channel introduction from neighboring southeast Asian countries, coexistence of multiple subtypes and continuous occurrence of new recombinants all lead to the most complex genetic characteristics of HIV-1 in Yunnan. As the pioneering ART implemented region, with a long history of ART use, Dehong Prefecture, undoubtedly, had a larger proportion of resistant strains among all epidemic ones in Yunnan. It has been well documented that the cross-border travelers were proved to be a key factor of the high infection rate and complex distribution of HIV in Yunnan [7, 8]. HIV-1 cross-border transmission and exchanges of various circulating subtypes were considered to be accelerated by this floating population. Moreover, migrations of travelers carrying resistant HIV-1 strains also bring great challenges to TDR surveillance work and AIDS treatment.

Located in the most southwestern Yunnan Province, Dehong prefecture has a shared border line of 503.8 km with Myanmar and is considered as the first station of illegal drug traffic from golden triangle region into China. Due to the special geographic location, the first endemic of HIV-1 in China was identified in Dehong, with the most HIV-1 prevalence and the complex distribution of HIV-1 subtypes or recombinants [9–11]. With the implementation of reform and opening-up policy in China, the cross-border travel and business became more frequent. More than 23 million travelers entered Yunnan province from Myanmar via Dehong port in 2017 (http://www.dh.gov.cn/swj/Web/_F0_0_28D00Q763C39JUSFW2UDF75NI6.htm).

Besides the continuous cross-border transmission of HIV-1 between China and Myanmar through travelers’ immigration, the prevalence of TDR HIV-1 strains among this huge cross-border population should be investigated too. Although it is obvious that HIV-1 infection rate of this population is much higher than other general groups based on our previous reports [8], the frequency of occurrence and types of transmitted drug resistance mutations (TDRMs) are still unidentified in this population. In current study, the plasma samples were collected randomly from entering travelers at Dehong port between 2003 and 2012. HIV-1 infection was determined and TDR mutations were further analyzed by sequencing on entire *pol* gene of HIV-1. The occurrence, types and molecular characteristics of these TDR strains will be further explored.

## Methods

### Participants

From 2003 to 2012, one of every five-hundred Myanmar travelers entering China at land ports of Dehong prefecture, Yunnan, was randomly sampled for HIV-1 test. The processes and methods of HIV-1 detection and laboratory confirmation have been described in our previous reports [8]. Totally, 22,699 travelers were required for HIV test, and 1163 were identified as HIV-1 infectors. Among them, 298 travelers who never received any antivirus therapy before (ART-naïve), were selected in this study. Those cases fall intoeach year irregularly, ranging from 27 (year 2011) to 56 (year 2008). The collected plasma samples were stored at − 80 °C until RNA extraction. Data of the social-demographic characteristics and risk behaviors for HIV infection were obtained from the enrolled participants via face-to-face interviews with trained medical personnel by administering a structured questionnaire.

### HIV-1 *pol* gene amplification and sequencing

HIV-1 RNA was extracted from plasma using the High Pure Viral RNA kit (Qiagen, Valencia, CA, USA). The partial HIV-1 *pol* gene fragment (HXB2:2147–3462) was amplified using the One-Step reverse transcription PCR kits (TaKaRa, Dalian, China) with primers of MAW26 (5′-TTGGAAATGTGGAAAGGAAGGAC-3′) and RT21 (5′-CTGTATTTCTGCTATTAAGTCTTTTGATGGG-3′). RT-PCR procedure was as follows: HIV-1 RNA denaturation at 65 °C for 30 s, addition of the reaction mixtures at 4 °C, incubation at 50 °C for 30 min, 94 °C for 2 min, then 35 cycles of 94 °C for 30 s, 55 °C for 30 s, and 72 °C for 2 min 30 s. Nested PCR was performed using 2× Taq PCR MasterMix (TaKaRa) with primers PRO-1 (5′-CAGAGCCAACAGCCCCACCA-3′) and RT20 (5′-CTGCCAGTTCTAGCTCTGCTTC-3′). PCR conditions were: 94 °C for 5 min, then 35 cycles of 94 °C for 30 s, 63 °C for 30 s, and then 72 °C for 2 min 30 s. Positive PCR products were visualized in agarose gel electrophoresis, and commercially sequenced by Biomed company (Beijing, China).

### Genotyping and drug-resistance mutations (DRMs) analysis

The obtained sequence of *pol* gene was assembled, edited and aligned as previously described [12, 13], and then submitted to NCBI GenBank to get accession numbers as MG787428-MG787459. The Stanford University HIV Drug Resistance Database HIVdb Program (version 8.4, https://hivdb.stanford.edu/hivdb) was used to investigate the presence of drug resistance mutations conferring resistance to Protease Inhibitors (PIs), Nucleoside Reverse Transcriptase Inhibitors (NRTIs), and Non-Nucleoside Reverse Transcriptase Inhibitors (NNRTIs) [14, 15]. Genotypes were determined in reconstructed maximum likelihood (M-L) tree using MEGA 7.0 software in the Kimura 2 parameter model with gamma distribution and invariant sites.

### Phylogenetic and recombination analyses of HIV drug resistant strains

To elucidate phylogenetic characteristics of these drug resistant strains, *gag-pol* gene sequences were obtained by using reverse transcription nested PCR as described before [12]. Phylogenetic analyses were performed using the M-L (Maximum likelihood) tree method based on the Kimura 2-parameter model with 1000 bootstrap replicates in MEGA version 7.0 [16]. The HIV-1 recombinant structure was screened using the Recombinant Identification Program available from the HIV database (www.hiv.lanl.gov/content/sequence/RIP/RIP.html). The suspected novel recombinant strains were subjected to boot scanning and informative-site analysis using SimPlot version 3.5.1 with boot-scan window sizes of 200 bases, a step size of 20 bases and 100 replicates [17].

## Results

### Demographic characteristics of recruited patients

Between 2003 and 2012, a total of 298 Burmese ART-naïve travelers with HIV-1 infection, who entered China at Dehong port in Yunnan province, China, were recruited in this study. Most of their age varieded from 21 to 40 (accounted for 76%) at their sampling time and 26 (10.44%) were female. Their occupations were long-distance drivers, businessman, migrant rural workers, hair salon workers, and tourists. Long-distance drivers, who transported goods shuttling between China and Myanmar, accounted for the majority in this population (Table 1). Unsafe sexual contact (12.80%) and intravenous drug use (33.20%) were major informed risk factors for HIV-1 infection; however, 135 infected travelers did not know how they got infected. *Pol* gene sequences (1.3 kb) were successfully obtained from 250 travelers. Phylogenetic analysis showed that CRF01_AE was the predominant HIV-1 subtype/CRF (142 cases, 56.8%), followed by subtype C (49 cases, 19.60%), subtype B (31 cases, 12.40%), CRF08_BC (9 cases, 3.60%), CRF07_BC (5 cases, 2.00%). 14 cases identified other CRFs, including CRF01B, CRF64_BC, and other types of B/C recombination (Additional file 1: Figure S1).

**Table 1:** Demographic characteristics of all recruited subjects for drug-resistance mutations detection

Characteristics	Cases(%)
Gender	
Male	224(89.56%)
Female	26(10.44%)
Years (mean, range)	
≤ 20	11(4.4%)
21–30	85(34%)
31–40	107(42.8%)
41–50	29(11.6%)
51–60	8(3.2%)
Occupations	
Long-distance Drivers	185(74%)
Businessman	14(5.6%)
Migrant Rural Workers	23(9.2%)
Hair Salon Workers	15(6%)
Tourists	9(3.6%)
Others^‡^	4(1.6%)
Subtypes	
CRF01_AE	142(56.8%)
C	49(19.6%)
B	31(12.4%)
CRF08_BC	9(3.6%)
CRF07_BC	5(2.0%)
Others	14(5.6%)
HIV risk factors	
Injecting drug users	83(33.2%)
Sex	32(12.8%)
Unknown	135(54%)

^‡^Includes several international students, medical staff and the individuals for whom we lack such information

### Prevalence of HIV drug resistant mutations

According to HIV Drug Resistance Database, 45 mutations associated with drug resistance were defined in 32 therapy-naïve Burmese travelers. Statistically, the rate of TDRs in this population was 12.80% (32/250). The annual TDRs rate was distributed irregularly and ranged from 0 (2005) to 21.88% (2004). Of 32 HIV-1 infected travelers with TDRs mutation, CRF_01AE (20 cases) was identified as predominant subtype/CRF, followed by subtype C (4 cases), CRF08_BC (2 cases), subtype B (2 cases) and other undetermined subtypes (4 cases). The TDRs rate was significantly different from the subtypes/CRF. Although 9 cases were infected with CRF08_BC in this population, 2 of them (22.22%) were proved to carry drug resistant sites. There was no TDRs observed with CRF07_BC.

**Fig. 1:**
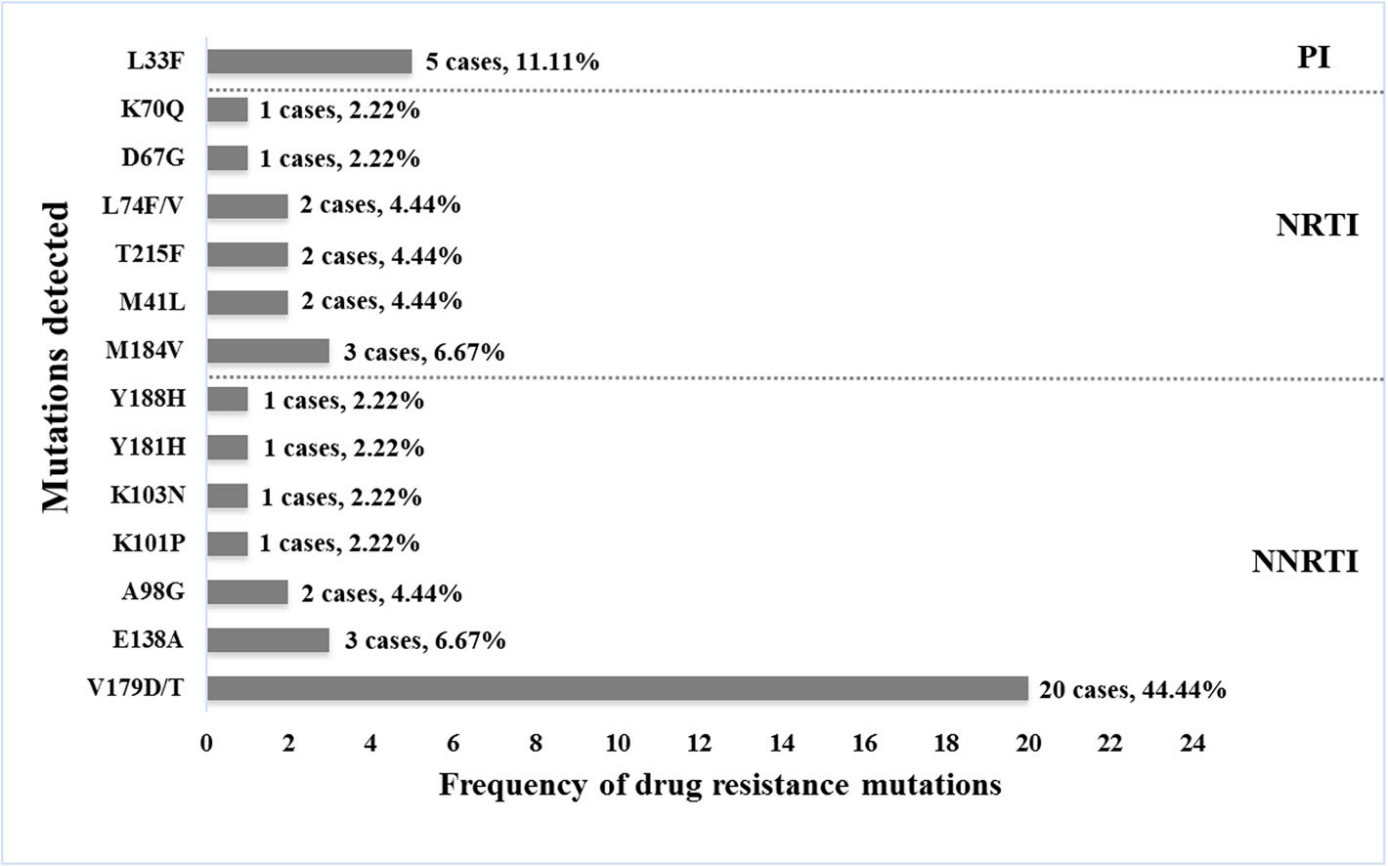
Forty-five mutations associated with transmitted resistance to any drug, defined according to the World Health Organization

**Fig. 2:**
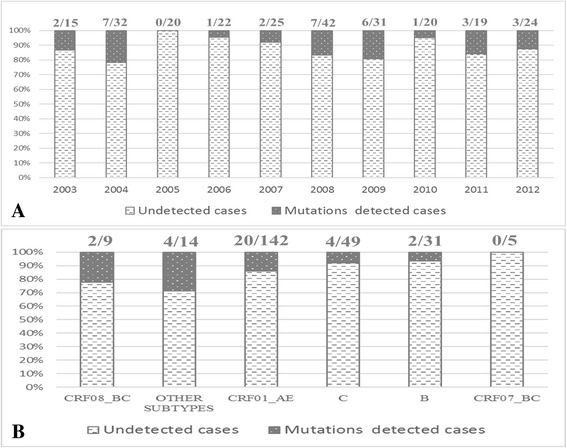
Trend of DRMs rate of Therapy-naïve Burmese entering travelers at Dehong port in the past decade (**a**) and the TDR frequency in different subtype (**b**)

In detail, mutations associated with resistance to NNRTI, NRTI, or PI were found in 29 (64.44%), 11 (24.44%) and 5 (11.11%) cases respectively (Fig. 1). For NNRTI resistance-associated mutations, V179D/T was identified as the major mutation (20 cases), and the other 6 types of mutation were E138A, A98G, K101P, K103 N, Y181H and Y188H. NRTI resistance-associated mutations found in this population included M184 V (*n* = 3, 6.67%), M41 L (*n* = 2, 4.44%), T215F (*n* = 2, 4.44%), L74F/V (*n* = 2, 4.44%), D67G (*n* = 1, 2.22%) and K70Q (*n* = 1, 2.22%) (Figure1). In addition, there was only one PI resistance-associated mutation found (L33F) in 5 cases. Furthermore, four (12.50%) subjects displaying DRMs had dual-class drug resistance mutations, including 2 cases to NRTI and NNRTI and other 2 cases to NNRTI and PI. No obvious annual change of DRMs occurrence was found from 2003 to 2012, and also no different DRMs distribution was observed in various subtypes/CRF (Fig. 2).

### Phylogenetic characteristics of the drug resistant strains

**Fig. 3:**
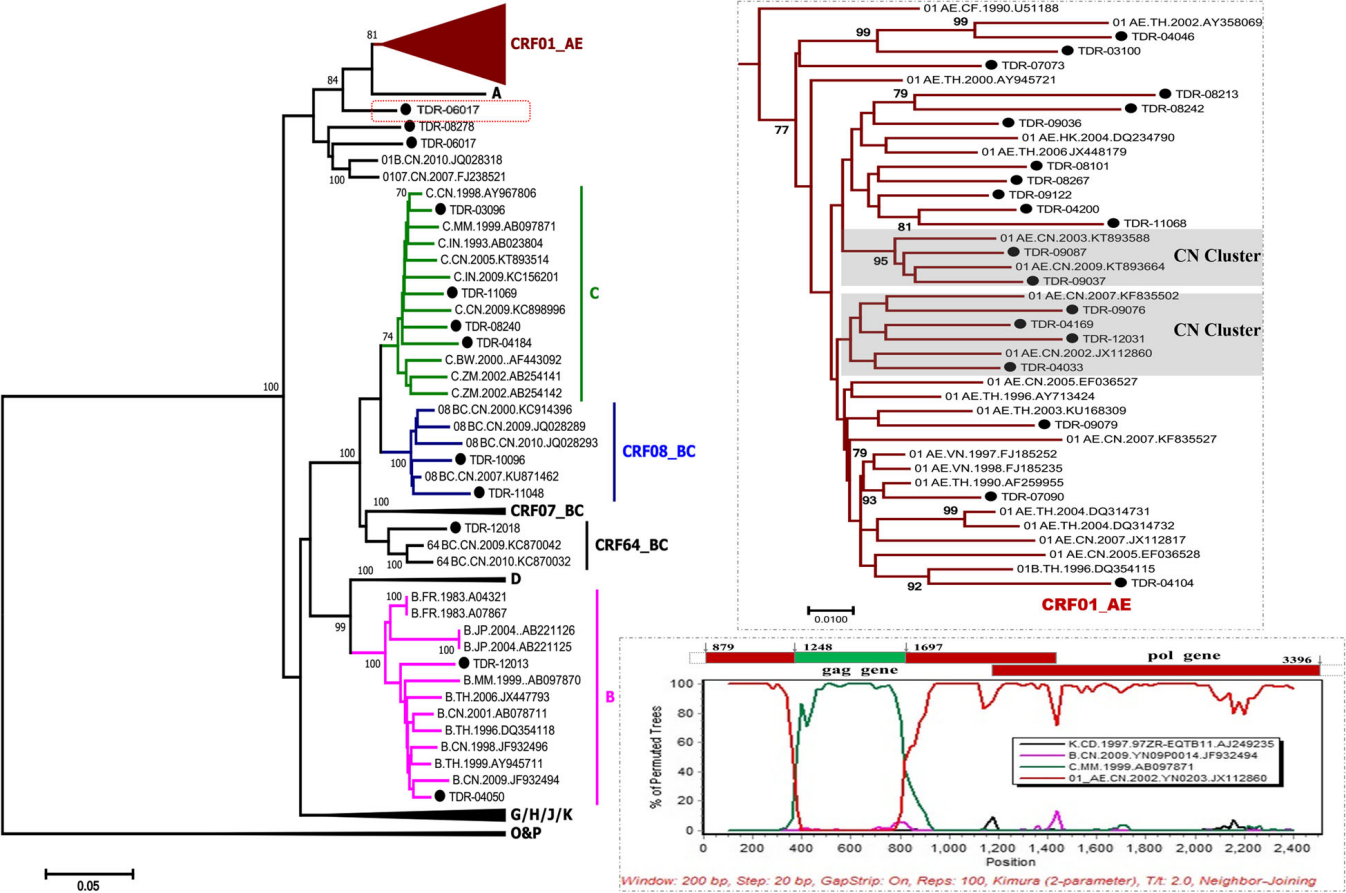
Maximum likelihood phylogenetic tree of the drug resistant strains in this study based on the sequence of HIV-1 gag-pol fragments and the recombinant structures of TDR-08269. The strains labeled with black circles were collected in this study. Strains that could not be precisely classified as known subtypes, CRFs, or URFs are shown in dashed red squares in the neighbor-joining tree

To elucidate phylogenetic characteristics of these drug resistant strains, sequences of *gag-pol* gene were used for phylogenetic analysis. As a result, 7 subtypes/CRFs of HIV-1 were found among these travelers infected with drug resistant strains (Fig. 3). All the used reference CRF08_BC strains were identified in China. The strains of subtype B and C in the current study were clustered together with the strains from China and neighboring countries, Thailand and Myanmar. The CRF01_AE strains, the most prevalent subtype among this population, showed close genetic relationship with reference strains from Southeast Asian countries (Thailand and Vietnam) and China. Two independent Chinese clusters were observed in the M-L tree, including 7 strains from this study. A Drug resistant strain, TDR-12018, was classified as CRF64_BC, which was firstly identified in IDUs (Injection drug users) from Dehong. The other two strains, TDR-06017 and TDR-08278, formed a tight cluster with the reference CRF01_B strains from Guangxi and CRF0107 from Jiangsu Province. However, TDR-08269 strain couldn’t be classified into any subtype/CRF. Further restructuring analysis showed it was inter-subtype of CRF01_AE and subtype C (Fig. 3) with small fragment gene of C inserted in CRF01_AE *gag-pol* gene.

## Discussion

Yunnan Province has been the key midpoint of traffic and business between south Asian countries and China. Dehong has been an important port in the business with Myanmar. The numerous cross-border population not only boosted rapid economic development but worked as carriers for HIV transmission. Thus, they were considered a ‘bridge population’ who may accelerate the exchange of virus genes between Yunnan and Myanmar. In our previous research, we have confirmed that HIV infection ratio (5.12%) of entering travelers at Dehong port is higher than any other ports in Yunnan [8]. This population, shaped by the specific characteristics of migration and HIV-1 infection susceptibility, has become a critical medium in the spreading of drug resistant strains. Therefore, it is of great necessity to investigate the prevalence of HIV infection and drug resistance strains among travelers from Myanmar.

In our previous study, a total of 1163 HIV infected Burmese travelers entering Yunnan at Dehong port have been detected. To address the prevalence of drug-resistance mutations among this bridge population, 298 travelers who never received any anti-virus therapy before, were randomly selected for this study. The young long-distance drivers and businessmen dominated in this population. Although the infection route of 173 cases is still unidentified, face-to-face interviewing has guaranteed the authenticity of obtained epidemiologic information and privacy of participants. Among the informed infection route, drug injection is still the main risk behavior due to the common syringe sharing. Phylogenetically, more than 6 subtypes/CRFs have been discovered, including the main CRF01_AE (142 cases, 56.8%), following by subtype C, subtype B. The HIV-1 subtype distribution is similar to that of Yunnan Province in previous reports [18, 19].

With the implementation of active ART, the occurrence of drug resistant mutation becomes more frequent, which results in more resistant strains transmitting among new HIV-1 infectors and therapy-naïve infectors [20]. A global study on TDR frequency shows the highest (12.9%) TDR rate is in North America and the lowest (4.2%) in Asia [21]. It has been documented that the prevalence of drug-resistance was 45.1% in the ART-failure individuals and 2.1% in the controlled therapy-naïve individuals in Yunnan province [22]. However, the prevalence of drug-resistance is still controversial in the controlled therapy-naïve individuals due to small recruited participants (2/94). In current study, the TDR rate is 12.8% among the therapy-naïve Burmese travelers entering Dehong port in Yunnan Province, which is far higher than the earlier reported level. The high prevalence of TDR mutation was also discovered in other regions of China, like Henan Province (14.3%) [23] and Beijing (11.96%) [24]. More than two decades of ART treatment in these areas could explain the higher TDR prevalence in Yunnan, Beijing and Henan, etc. However, the prevalence of TDR among the bridge population is obviously higher than any other groups in Yunnan, even compared with homosexual group in Kunming (4.6%) [25], and general population in Dehong (< 5%) [26].

One type of PI mutations (L33F), six types of NRTI mutations and seven types of NNRTI mutations have been identified in this population. The major TDRMs observed in this study have been reported in a previous study of HIV Drug Resistance among ART-failure individuals in Yunnan Province, which figured out mutations such as M184 V/I, K103 N, V106A, Y181C and G190A were common [22]. Notably, the similar prevalence of TDRMs has also been found among therapy-naïve HIV patients in neighboring countries Vietnam [20] and Thailand [27]. It suggested a close correlation between the TDRMs from this population and the drug resistant mutants generated during the course of ART. Furthermore, M184 V is the most frequently detected NRTI resistance mutation, which was also found in previous studies [28, 29]. L33F and V179D/T are the types of mutation that confer potential low-level resistance. Even considering the previous report of occurrence in Hebei and Beijing, the mutation rate of V179D/T up to 6.92% is still an astonishing level [30, 31]. Despite of the previous report stating the possible relationship between TDRMs and HIV-1 subtype [32], there is little evidence shown in this study supporting this statement, possibly due to the relatively smaller sampled group in the investigation. However, DRMs analysis was not carried out on the mutations of integrase inhibitors in this study, which were also significant class of resistant mutations.

Phylogenetic analysis on *gag-pol* gene of all resistant strains has also been performed to identify their possible origins in this study. The results demonstrate that the Dehong epidemic strains share a very close genetic relationship with the strains from Yunnan, neighboring provinces (Guangxi) and countries (Thailand, Myanmar, etc). The high prevalence of TDR strains in these regions is reasonable considering the long and earliest history of ART implementation. The floating population, whose inherent mobility determines the multi-regional sources of the epidemic resistant strains, is considered as the highly possible TDR strains carrier in further spreading to broader range. Thus, it is critical and fundamental to monitor their drug-resistance proceeding to prevent them from further transmission of drug resistant strains.

## Conclusions

This study focuses on the occurrence of TDR and its phylogenetic characteristics in Burmese travelers. The prevalence of TDR among this group is as high as to 12.8%, far in excess of general population and reported level in China. The high similarities of drug resistant mutations between this population and ART failure individuals, imply a very close genetic distance between our resistant strains and epidemic strains from Yunnan and its surrounding areas. Based on these similarities, we speculate that HIV-1 drug resistant mutants are transmitted and spreading from the ART-undergoing populations to the new infectors.

## Additional file

Additional file 1: **Figure S1.** Maximum likelihood method was chosen for phylogenetic analysis and constructed phylogenetic tree using all the *pol* gene sequences. (PDF 133 kb)

## Abbreviations

AIDS: Acquired immune deficiency syndrome
ART: antiretroviral therapy
DRMs: drug-resistance mutations
HIV-1: Human immunodeficiency virus type 1
IDU: Injection drug user
M-L: Maximum likelihood
NNRTIs: Non-Nucleoside Reverse Transcriptase Inhibitors
NRTIs: Nucleoside Reverse Transcriptase Inhibitors
PIs: Protease Inhibitors
TDR: transmitted drug resistance
TDRMs: transmitted drug resistance mutations
