# Supplementary figures and images for: High prevalence of HIV-1 transmitted drug resistance among therapy-naïve Burmese entering travelers at Dehong ports in Yunnan, China
Source: BMC Infect Dis. 2018 May 8;18:211. doi: 10.1186/s12879-018-3130-9 (PMC5941624; doi:10.1186/s12879-018-3130-9)

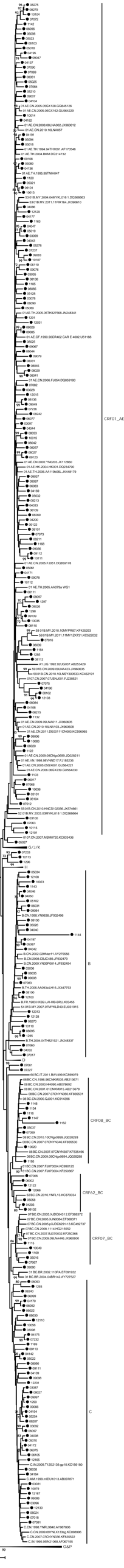

Supplement: Supplementary file 1 — Figure S1. Maximum likelihood method was chosen for phylogenetic analysis and constructed phylogenetic tree using all the pol gene sequences. (PDF 133 kb) [file 12879_2018_3130_MOESM1_ESM.pdf]
